# In Silico Validation of D7 Salivary Protein-derived B- and T-cell Epitopes of Aedes aegypti as Potential Vaccine to Prevent Transmission of Flaviviruses and Togaviruses to Humans

**DOI:** 10.6026/97320630013366

**Published:** 2017-11-30

**Authors:** Sathish Sankar, Mageshbabu Ramamurthy, Balaji Nandagopal, Gopalan Sridharan

**Affiliations:** 1 Sri Sakthi Amma Institute of Biomedical Research, Sri Narayani Hospital and Research Centre, Sripuram, Vellore - 632055, Tamil Nadu, India;

**Keywords:** mosquito salivary protein, D7, Epitope, MHC, Flaviviruses, Togaviruses

## Abstract

Mosquito (Aedes aegyptii) salivary proteins play a crucial role in facilitating viral transmission from vector-to-host due to their role in facilitating the "blood meal" of the vector. Three main proteins, D7, aegyptin and Sialokinin play a role in this process. Using in-silico programs, we identified B- and T-cell epitopes in the mosquito salivary proteins D7 long and short form. T-cell epitopes with high affinity to the most prevalent HLA MHC class-I supertypes among different population groups was chosen. It is our postulate that these epitopes could be successful in eliciting B and T cell responses, which would decrease the vector blood meal efficiency and hence protect against host infection by certain viruses. These include causative agents like Dengue viruses, Chikungunya virus, Zika and Yellow fever viruses. These viruses are of major public health importance in several countries in the Americas, Asia and Africa. Experimental evidence exists in previously published literature showing the protective effect of antibodies to certain salivary proteins in susceptible hosts. A novel approach of immunizing humans against the vector proteins to reduce transmission of viruses is now under investigation in several laboratories. We have identified the following two B cell epitopes LAALHVTAAPLWDAKDPEQF one from D7L and the other TSEYPDRQNQIEELNKLCKN from D7S. Likewise, two T cell epitopes MTSKNELDV one from D7L and the other YILCKASAF from D7S with affinity to the predominant MHC class-I supertypes were identified towards evaluation as potential vaccine.

## Background

Aedes aegypti is the primary vector of several medically relevant arboviruses including dengue virus, chikungunya, Zika and Yellow fever viruses. The vector transmits pathogens by inoculating infected saliva into host skin during probing and feeding. Ae. aegypti saliva contains over one hundred unique proteins. Salivary proteins are majorly responsible for the interaction between mosquitoes and vertebrate host during blood feeding that includes inhibition of blood clotting, platelet aggregation and vascular contraction [1]. Three main proteins, D7, aegyptin and Sialokinin are likely targets for host's protective immunity [2, 3].

Ae. aegypti salivary gland proteome analysis using highresolution mass spectrometry showed 29 proteins involved in immunity-related pathways. It has been postulated that such proteomic data enable future vaccine design and development of virus-blocking strategies and identification of novel molecular targets in the mosquito vector Ae. aegypti [4]. Among these, the D7 protein along with a few others can elicit host antibody response [5]. It is hypothesized that antibodies to proteins to D7 may offer protection to the host. D7 proteins function as scavengers of biogenic amines or other hemostasis agonists and are the most abundant subfamily of salivary proteins [6].

Major histocompatibility complex (MHC) and T cell receptors play a crucial role in mounting an efficient adaptive immune response. The MHC proteins bind and present short antigenic peptides on the infected cell surface. Commonly virus-derived peptides are presented to CD8 cytotoxic T cells as peptide-MHC complexes, for recognition by the T- cell receptors (TCR). This results in a series of events required for initiation and regulation of immune response [7]. As viruses can infect any nucleated cell, Almost all cells express MHC class I molecules, though the expression level varies from one cell type to the other [8]. This is called Class-I restricted antigen recognition on antigen presenting cells-I (APC-I). B cells too have a similar system wherein the monomeric immunoglobulin on B cell surface recognize antigen, referred as B cell receptor (BCR). This recognizes epitopes in the context of MHC class II molecules and on CD4 T Helper cells. The BCR molecules recognize epitopes on another group of APC defined as APC-II, which includes B cells, macrophages and dendritic cells [9].

The incidence and geographic range of arboviral infections have increased dramatically in recent decades. There is no specific effective drug treatment against most of these viruses. The existing licensed vaccine for Dengue viruses has only 60% efficacy and response to serotype 2 is variable raising questions on susceptibility of vaccinees to severe complications of dengue fever [10].

Several vector control measures have been attempted with varying degrees of success to slow down transmission [11]. Conventional approaches of vaccine development (pathogen specific) do not seem to have made much impact against dengue fever but is successful against Yellow fever virus. More recently, bioinformatics-based approaches in identifying B- and T- cell epitopes predicted from the genome sequence databases are possible. Advanced B-cell epitope mapping programs that are developed with abundant datasets provide information for the development of B-cell epitope based vaccines. Likewise, there has been some progress in developing T-cell epitope MHC class I matched designer vaccine for different geographical regions [12]. Peptide fragments that serve as the cytotoxic T lymphocyte (CTL) epitopes are processed from virus by the proteasome and then are transported to the endoplasmic reticulum through transporter associated with antigen processing (TAP) and then loaded onto the MHC class I molecule [13].

Here, we aimed to identify B- and T-cell epitopes that are specific to D7L and D7S salivary protein of Ae. aegypti transmitting arboviral infections in humans. The D7 proteins were modeled to generate 3D structure. T-cell epitope candidates of Ae. aegypti D7 are restricted to MHC Class I HLA allele supertypes were studied. HLA alleles are highly polymorphic in ethnic populations but are groupable into a few supertypes. These HLA supertypes have similar peptide sequences but with distinct antigen-binding repertoire [14] Proteasomal clevage, TAP efficiency, immunogenicity and the population coverage of specific HLAs were analyzed. B-cell epitope was identified from D7L and D7S towards design of generic vaccine against mosquito transmitted flaviviruses and togaviruses.

## Methodology

In order to carry out the in silico analysis, the steps taken are listed in the chronological order of the programs used for each action.

### Sequence retrieval

The amino acid sequence of salivary protein D7 long form (D7L) and short form (D7S) of Aedes aegypti was retrieved from GenBank database as amino acid sequence (accession number: AF420272 and DQ440049). The proteins are 332 and 158 amino acids in length respectively. Two long and four short allelic forms have been expressed in the salivary tract. Both long and short form D7 proteins and the 34 kDa salivary protein are found in Ae. aegypti salivary glands. The shorter D7 proteins are found in both anophelines and culicines. We chose one each from long form and short form to identify potential candidate vaccine B- and Tcell epitopes. This was done because all Aedes species possess the selected allelic form, which was fully characterized and available in the GenBank database.

### Prediction of B-cell epitopes

Linear B-cell epitopes (20mer) for D7L and D7S were identified using three different programs: ABCpred, LBtope, and BepiPred 1.0 server. ABCpred server [15] was used to predict immunodominant, immunogenic linear B-cell epitopes from the D7L and D7S protein sequences. This program was developed based on artificial neural network (machine learning technique) using fixed length patterns. The program allows analysis for up to 20-mer lengths. We used the default threshold limit of 0.51 was set and the epitope window length of 20 was used.

LBtope prediction server [16] was used to identify linear B-cell epitopes from the D7L and D7S of Aedes aegypti amino acid sequences. This program is based on support vector machines (SVM) built with more datasets compared to ABCpred program. LBtope Fixed (original dataset) was used to identify fixed length (20-mers) of epitopes from each query sequence. The program selected epitopes with the percentage of probability more than 60 (SVM score >0.3). This program thus used will generate information on any epitope with more than 60% prediction probability for binding level to MHC molecules.

Linear B-cell epitopes were identified also using BepiPred 1.0 server [17] with threshold score of 0.35. This program uses a combination of a hidden Markov model and a propensity scale method. The residues with scores above the threshold are predicted to be part of an epitope.

### Protein modeling

Protein structure modeling was carried out by four programs to examine the protein folding. This is understood to be the basis for epitope presentation especially to B cells. Three-dimensional structure modeling of D7L and D7S proteins was carried out using I-TASSER [18], Swiss-Model [19], Phyre2 [20] and Raptor-X [21] programs. We used these 4 different programs to generate the best model because there was no closely matching 3D structure available in protein database (PDB). Among a list of structures generated by the programs, one best model was selected each from 4 programs based on its distinctive scores. ITASSER model was chosen based on confidence score (C-score) that is calculated from template modeling (TM)-score and rootmean- square deviation (RMSD) to measure the accuracy of structure modeling. C-score is a measure of quality of predicted models and ranges from -5 to 2. High value of C-score indicates high model confidence. Swiss-model generated structure was validated using a composite scoring function that includes QMEAN and GMQE (Global Model Quality Assessment). QMEAN Z-scores indicate better agreement between the model structure and experimental structures of similar size. GMQE score reflects the expected accuracy of a model built with that alignment and template expressed as values between 0 and 1. Ramachandran plots were generated for the models selected by the 4 programs using RAMPAGE program [22]. The number of residues in favored region, allowed regions and as outliers was analyzed from the plots. ERRAT and Verify_3D programs were used to further validate the quality of the models using SAVES (The Structure Analysis and Verification Server, version 4) online server program [23]. The structures were visualized and analyzed with PyMOL.

### Prediction of T-cell epitopes from the D7L and D7S protein of Aedes aegypti with affinity to Class I MHC molecules

The amino acid sequence of D7L and D7S were analyzed for the determination of their respective T-cell sequences binding to MHC-class I HLA supertype representatives (n=27) and qualified as T-cell epitopes. We selected the most common HLA alleles (a reference panel of 27 alleles). The reference sets was based on the following criteria: 1) the most common specificities in the general population, based on data available from public databases (DbMHC and www.allelefrequencies.net); 2) representative of commonly shared binding specificities (i.e., supertypes). The reference sets for class I provide > 97% population coverage [24, 25]. The selected alleles were based on the percentage chance of haplotype expressed in an individual identified from HLA matchmaker program available at http://www.epitopes.net/. The epitopes that had high binding levels (high affinity) were selected for further evaluation of proteasomal cleavage and TAP prediction.

Using the amino acid sequences as the input, T-cell epitopes that bind to MHC Class I were predicted using BepiPred MHC-I binding prediction tool. This program predicts binding of peptides to any MHC molecule of known sequence using a combination of Artificial neural network (ANN), Stabilized matrix method (SMM), SMM with a Peptide:MHC Binding Energy Covariance matrix (SMMPMBEC), Scoring Matrices derived from Combinatorial Peptide Libraries (Comblib_Sidney2008), Consensus, NetMHCpan, NetMHCcons, PickPocket and NetMHCstabpan. The median percentile rank of the methods used is reported as the representative percentile rank. Therefore a lower number indicates higher affinity. The default threshold for good binding in terms of % rank (≤1), was used in our study. The epitopes of 9-mer and 10-mer lengths were derived. Epitopes with binding affinity with the set threshold alone were selected and used for further analysis.

### Prediction of proteasomal cleavage

MAPPP (MHC-I Antigenic Peptide Processing Prediction) program [26] was used to predict proteasomal cleavage. The program generates a probability for the cleavage of each possible peptide from a protein by the proteasome in the cell and the probability is based on a statistic-empirical method. The algorithms in the program were earlier implemented in FRAGPREDICT. Minimum possibility for cleavage after a single residue and for cleavage of a fragment was set to default value of 0.5.

### Prediction of TAP efficiency

TAPPred server program [27] was used to predict the candidate epitope(s) based on the predicted processing of the peptide(s) in vivo, the transporter of antigenic peptides (TAP) proteins' transport efficiency. The prediction approach used in this study was cascade SVM, a prediction that is based on the sequence and features of amino acids and their properties that include polarity, charged residues, volume, aromatic residues and flexibility.

### Prediction of immunogenicity

The identified epitope(s) were used to predict class I immunogenicity using IEDB analysis resource program [28]. This tool uses amino acid properties as well as their position within the peptide to predict the immunogenicity of a peptide-MHC complex.

### HLA distribution

The global distribution of selected HLA alleles to which the identified epitopes bind was analyzed. The Allele Frequency Net Database [29] provides a central source, for the storage of allele frequencies from different polymorphic areas in the Human Genome of global population. Classical allele frequency tool was used to search allele frequency for all geographical regions as available in the database. It includes Australia, Europe, North Africa, North America, North-East Asia, Oceania, South and Central America, South Asia, South-East Asia, Sub-Saharan Africa and Western Asia. Countries listed in Europe alone were further classified as Northern, Eastern, Western and Southern Europe based on public database [30]. The allelic frequencies (in decimals) were identified and the average value of each category was calculated and analyzed. Allele Frequency in decimals refers to the total number of copies of the allele in the population sample (Alleles / 2n) in decimal format that represents frequencies of large datasets (e.g. where sample size > 1000 individuals). The average allelic frequency values for each geographical region was used to prepare an excel spreadsheet and the program was used to generate frequency of HLA supertype distribution graphs.

## Results

### B cell epitope prediction

ABCpred server was used with the set epitope length of 20 and threshold limit of 0.51. The program resulted in identification of 27 epitopes for D7L with more than the threshold value chosen. Epitope LGWKLEPSDDQATQCYTKCV was identified with highest score of 0.94. Higher score of the peptide indicates the higher probability to be as epitope. Other epitopes ranged from 0.9 to 0.67. SVM based LBtope Fixed program identified epitopes of 20-mers lengths from D7L and D7S. Epitopes with the percentage of prediction probability ≥60 (SVM score >0.3) were selected. The program predicted a total of 313 overlapping epitopes. LBtope also identified the 27 epitopes identified by ABCPred program. Of these, 12 epitopes had less LBtope score (<0.3), though had high ABCpred score (0.67 to 0.9) and not analyzed further. The remaining 15 epitopes that are generated by both LBtope (0.33 to 1.06) and ABCpred (0.72 to 0.94) are shown in Table 1.

Linear B-cell epitope analysis using BepiPred 1.0 server resulted in the identification of two 20-mer epitopes KGESSKKYYQEKGIKIKQKG and KLKPYSRSDVRKQVDDIDKI for D7L. The two epitopes were also identified by LBtope program and not by ABCpred program. The former also had very low prediction score for MHC class II binding. Based on the overall results, epitope LAALHVTAAPLWDAKDPEQF (SVM score 1.07 and ABCpred score 0.76) was considered as best suitable candidate B-cell epitope specific for D7L protein.

For D7S protein sequence, ABCpred program resulted in 11 epitopes with scores ranging from 0.78 to 0.9 and LBtope program resulted in 139 overlapping epitopes, of which 46 epitopes had score more than 0.3 threshold limit. Both ABCpred and LBtope programs identified Epitopes TSEYPDRQNQIEELNKLCKN, KNIYDPPRNAMNYLDCIALR and FIRKREPKFFNVFHCRGITL with scores above the threshold limit. BepiPred 1.0 server program did not detect any 20-mer epitope for D7S. Based on the above results, epitope TSEYPDRQNQIEELNKLCKN (SVM score 0.74 and ABCpred score 0.69) was considered as best suitable candidate B-cell epitope specific for D7S protein (Table 2).

### T cell epitope prediction

Class I MHC T-cell epitopes binding to each of the supertype HLAs were predicted individually for the D7L and D7S sequences. The IEDB MHC-I binding prediction program resulted in identification of 168 epitopes for D7L and 99 epitopes for D7S. Among these, only 13 epitopes survived proteasomal cleavage as predicted by MAPPP program, of which four epitopes showed promiscuous binding with more than one HLA supertype. The results are shown in Table 3. Among the 99 epitopes for D7S, only 12 epitopes survived proteasomal cleavage as predicted by MAPPP program, of which four epitopes showed promiscuous binding with more than one HLA supertype (Table 4).

The selected epitopes matched to one or more 13 supertype HLA alleles (HLA-A*01:01, HLA-A*23:01, HLA-A*24:02, HLA-A*30:01, HLA-A*32:01, HLA-A*33:01, HLA-A*68:01, HLA-B*08:01, HLAB* 15:01, HLA-B*40:01, HLA-B*44:02, HLA-B*57:01 and HLAB* 58:01). The geographical distribution of the 13 HLAs in terms of allelic frequency was analyzed. The results are shown in Figure 5 and Figure 6. Based on the analysis, HLA-A*24:02 was found predominant in almost all parts of the globe followed by HLAA* 01:01 and HLA-B*08:01. In all four parts of Europe, HLAA* 01:01 was found widely distributed in higher frequency.

Based on the prediction results obtained and HLA distribution, T-cell epitope, MTSKNELDV from D7L and epitope YILCKASAF from D7S were considered suitable candidate vaccines for global use.

### Protein structure modeling

The three-dimensional (3D) modeled structure for the D7L and D7S proteins were generated by I-TASSER, Swiss-Model, Phyre2 and Raptor-X programs. Each program generated a list of models. One model from each program was selected based on the scores. Among the structures predicted by I-TASSER, the C-score of D7L model 2 and D7S model 1 was -0.21 and -0.74 respectively and therefore selected. In Swiss-model, the D7L structure that obtained a high GMQE score of 0.69 and a QMEAN Z-score of - 2.74 was selected. The D7S model obtained a GMQE score of 0.43 and a QMEAN Z-score of -2.31. Phyre2 generated models were selected based on confidence score and identity. RaptorX generated only one model each for D7L and D7S.

**Figure 1:**
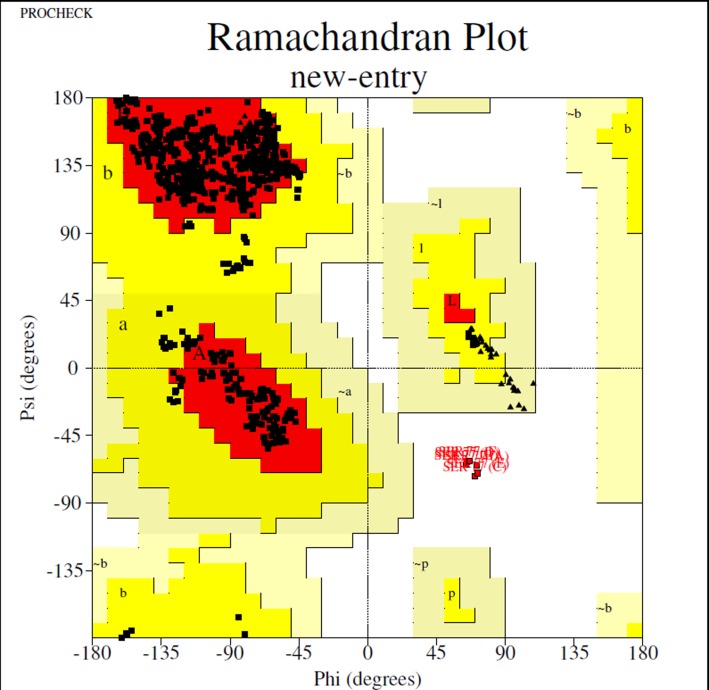
Ramachandran plot for D7L plot generated using RAMPAGE program

In the case of D7L, Ramachandran plot generated by RAMPAGE, the number of amino acid residues in the favored region was higher in the model generated by Phyre2 (97.3) followed by the one generated by Swiss-model (95.5) (Figure 1). It is accepted for the Ramachandran plot analysis when 90% or more of the residues exist in the "core" region of the protein. Here, one would expect to have the best stereochemical quality of a protein structure. I-TASSER generated model had the least number of residues (83.9). The program Verify_3D determines the compatibility of an atomic model (3D) with its own amino acid sequence (1D) by assigning a structural class based on its location and environment (alpha, beta, loop, polar, nonpolar etc) and comparing the results to good structures. At least 80% of the amino acids that score ≥ 0.2 in the 3D/1D profile were considered passed by the program. ERRAT is based on comparison of plots of an initial model and a final model. Regions of the structure with values more than 95 are expected to have only 5% error value. Though all the models passed the Verify_3D score, the ERRAT quality factor of the Swiss-model generated structure alone was qualified.

**Figure 2:**
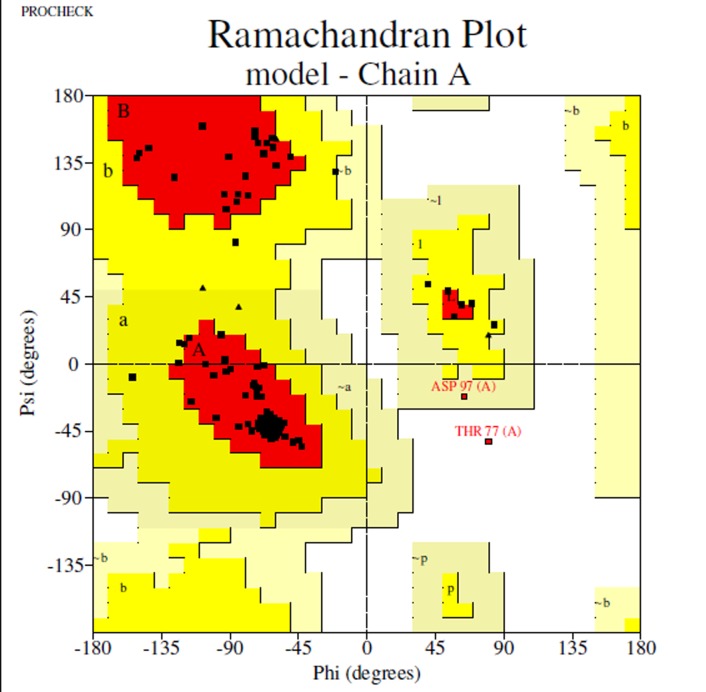
Ramachandran plot for D7S plot generated using RAMPAGE program

In the case of D7S, Ramachandran plot generated by RAMPAGE, the number of amino acid residues in the favored region was higher in the model generated by Phyre2 (98.3) followed by the one generated by Swiss-model (93.8) (Figure 2). Only the Swissmodel generated model passed the Verify_3D score and scored highest in ERRAT quality score (Table 5).

**Figure 3:**
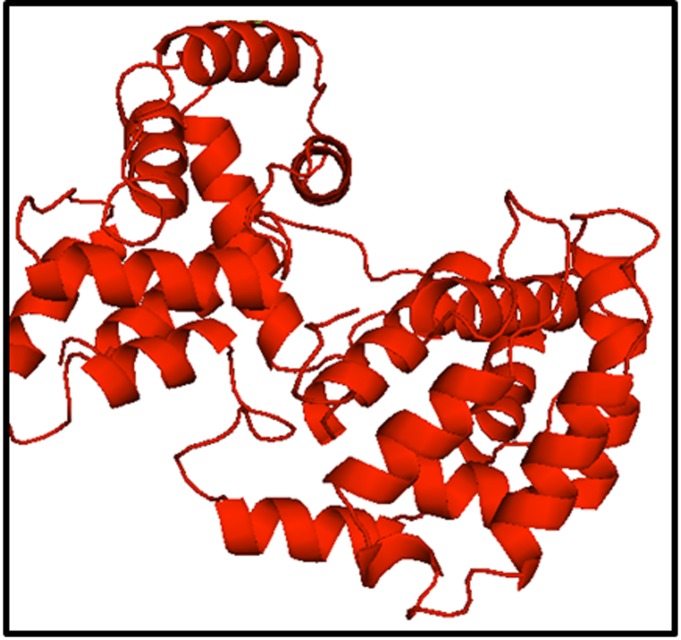
Swiss-model generated for D7L protein. This model compared to other three models (I-TASSER, Phyre2 and Raptor X), had high ERRAT quality factor and passed the Verify_3D score

**Figure 4:**
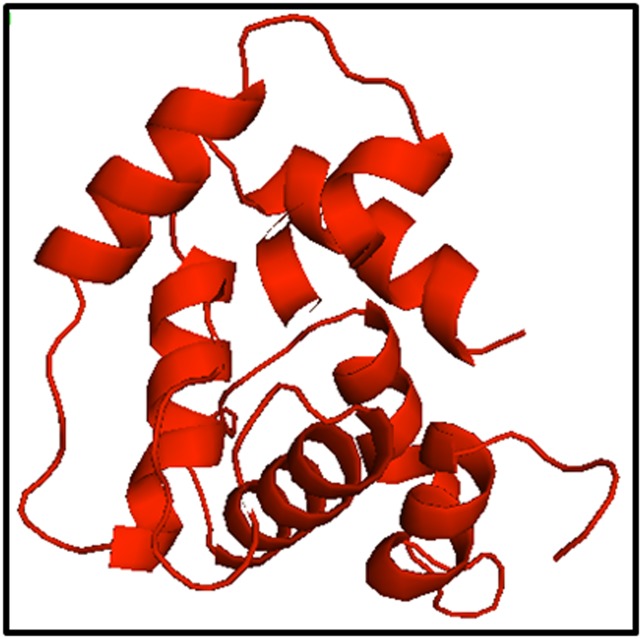
Swiss-model generated for D7S protein. This model compared to other three models (I-TASSER, Phyre2 and Raptor X), had high ERRAT quality factor and passed the Verify_3D score.

**Figure 5:**
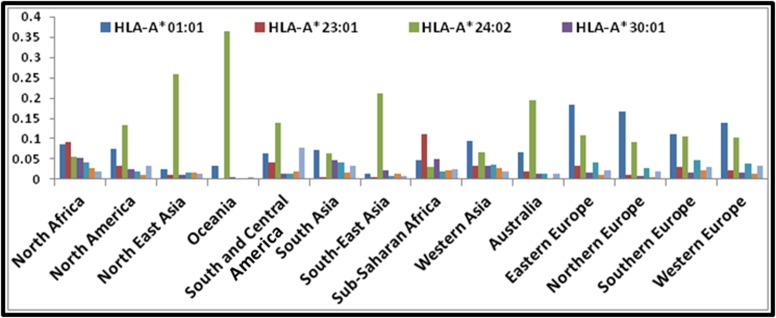
Geographical distribution of HLAs (HLA-A) allelic frequency (number of allelic copies in the population sample) in decimals. X -axis indicates geographical regions; and y-axis indicates allelic frequency in decimals

**Figure 6:**
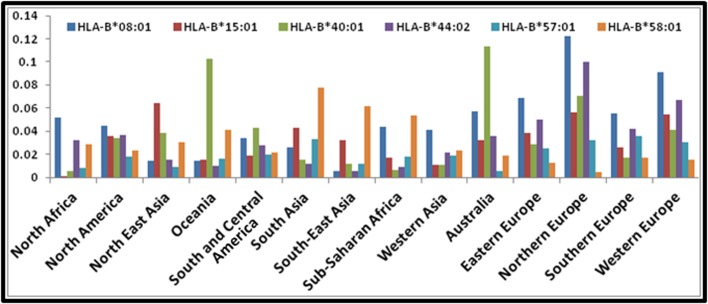
Geographical distribution of HLAs (HLA-B) allelic frequency (number of allelic copies in the population sample) in decimals. X -axis indicates geographical regions; and y-axis indicates allelic frequency in decimals

Best D7L and D7S models were selected based on the individual prediction program scores, number of residues in the favored region, ERRAT quality factors and Verify_3D scores. Based on the results, the D7L and D7S models generated by Swiss-model were considered best. The models were analyzed using Pymol program (Figure 3 and Figure 4).

## Discussion

The present study involves a comprehensive search for suitable B- and T-cell epitope predicted by bioinformatic tools in the mosquito salivary protein D7L and D7S. Analysis of the geographic distribution of HLA supertypes for the purpose of binding strength analysis of T cell epitopes was also carried out. B and T cell epitope identification in this fashion of vector proteins as potential vaccine was the goal. We identified B- and T-cell epitope as candidate vaccine cocktail from D7 long form and short form salivary proteins of Ae. aegypti. In addition, we also carried out protein modeling to evaluate our findings.

Vector-borne diseases account for more than 17% of all infectious diseases, causing more than 1 million deaths annually. More than 2.5 billion people in over 100 countries are at risk of contracting dengue alone [31]. The greatest health risk of arboviral emergence comes from extensive tropical urbanization and the colonization of this expanding habitat by the highly anthropophilic (attracted to humans) mosquito, Ae. aegypti. This is the primary vector for Dengue fever, Chikungunya, Zika and Yellow fever [32, 33, 34]. Except for Yellow fever prevention by vaccine, there is an ongoing search for the other viruses. It is vital to undertake a contemporary research approach to prevent other life threatening infections, which are Ae. aegypti -borne likely to emerge in the future.

Ae. aegypti injects saliva primarily into host extravascular dermal spaces for feeding. The mosquitoes express unique proteins, the D7 family belonging to odorant-binding protein superfamily that has been recognized to be specifically expressed in the salivary glands of adult Aedes mosquitoes. There exist two D7 subfamilies, long form (27-30 kDa) and short form having (15-20 kDa) of the salivary gland proteins. Transcripts of the D7 gene family are widely found in mosquito sialotranscriptomes. These proteins have a role in affecting blood clotting, platelet aggregation and vascular contraction [1]. This is to overcome host barriers, including the physiological responses elicited by hemostatic and inflammatory systems during a mosquito "blood meal". Certain protein digestive enzyme transcripts such as serine protease have been shown to enhances West Nile virus and Dengue virus pathogenesis in a mouse model, possibly by modulating T-cell responses, indicating the importance of salivary proteins for enhancing virus infectivity during transmission [35, 36].

Ribeiro et al. [37] identified transcripts (including splice variants and alleles) from adult Ae. aegypti female salivary gland protein analysis libraries. D7 members of the mosquito proteome were found over expressed in female salivary gland. The ratios of salivary gland transcriptome to whole body transcriptome were over 1,500, of the same order. Uniquely female gland expressed proteins such as aegyptin, salivary serpins or the D7 members. People living in endemic areas had specific antibodies against the D7 protein but not in healthy non-exposed individuals or infants [38]. It was postulated that the D7 protein initiates a specific antibody response in people living in endemic areas due to the frequent exposure to Ae. aegypti saliva. In a recent study [39], D7 protein was shown to interact directly with DENV virions and DENV envelope recombinant protein. Our postulate is, however, that D7 protein-derived peptide vaccination could produce antibodies blocking the function of D7 protein of the mosquito thus interfering with the "blood meal" and hence inhibit virus transmission. Anti-salivary protein antibodies were analyzed as a measure of the level of human- Ae. aegypti mosquito contact [40].

It has been shown that preexposure to uninfected mosquitoes could reduce parasitic load in the liver of the animals (BALB/c mice) infected with malarial parasites. Also, exposure to sequential bites of uninfected mosquitoes altered the immune response favorably with increased participation of T-helper cells [41]. It is potentially possible that JE virus, which could be transmitted, by Culex and Aedes species [42] may also be interfered with human host immune response to D7 short forms. Indeed, in this context a concern would be the role of enhancing antibodies in the increased virulence of Dengue virus infection resulting in complications like Dengue hemorragic fever and Dengue shock syndrome. It is understood that heterotypic antibodies that are not neutralizing serve to enhance virus infection of monocytic cells resulting in a "cytokine storm", especially in Dengue virus 2 infection in a person previously infected with another type [43]. It could be postulated that antibodies to D7, which is a mosquito salivary protein, would reduce the virus inoculum from the mosquito into the human host thus circumventing the apprehension of enhancing antibodies.

Two long and four short allelic forms have been expressed in the salivary tract [44]. Both long and short form D7 proteins and the 34 kDa salivary protein are found in Ae. aegypti salivary glands [45]. The shorter D7 proteins are found in both anophelines and culicines. In sandfly vector's salivary gland, a protein named PpSP15 was found to be similar to short D7 and probably related to the common ancestor [46]. We analyzed both long and short forms of D7, for which protein database and amino acid sequence information was available, for epitope prediction and modeling. The short form of D7 is seen in both Aedes and Culex species, which transmit flaviviruses and togaviruses.

There is no vaccine available to protect against Zika and Chikungunya viruses. The most recent tetravalent live-attenuated Dengue virus vaccine candidate showed a overall low immunogenic efficacy rate in a recently published phase 2b clinical trial [47]. Linear epitopes for DENV-2 and DENV-3 and discontinuous epitopes for DENV-2 and DENV-3 were predicted from the envelope proteins using ElliPro and the protein was modeled by using Phyre2 V 2.0 server [48]. Dikhit et al [49] proposed combined MHC class-I epitope vaccine strategy for Zika virus. T- and B- cell epitope vaccine candidates against ZIKV envelope protein have been predicted recently [50] using Immune Epitope & ABCPred databases.

In our analysis of the proteins, we carried out protein structure modeling by four programs to examine the protein folding which forms the basis for epitope presentation especially to B cells. The Swiss-model compared to other three models (I-TASSER, Phyre2 and Raptor X), had high ERRAT quality factor and passed the Verify_3D score. This validates that the protein-derived epitopes under scrutiny are likely to be potent immunogens. One best Band T- cell epitope each from D7S and D7L was selected and presented in this study. This prediction is enhanced by looking at its ability to transport efficiently without getting cleaved by the human proteasomes. In the prediction of class-I MHC binding Bcell epitopes, three programs, ABCPred, LBtope and BepiPred server were used in order to screen peptides predicted at least more than one program. In the prediction of protein 3D structure, four different programs were used to choose a best model with high prediction probability scores.

Towards malaria vaccine development, transmission blocking malaria vaccines, which induce antibodies that target the sexual stages of the parasite, has become a focus. Alanyl aminopeptidase N [51] and glutamate-rich protein formulations [52] of P. falciparum found produce high antibody titres recognizing the native protein in macrogametes/zygotes thus blocking the transmission. The efficacy of the filarial epitope protein, a chimera of selective epitopes, was tested in a murine model of lymphatic filariasis with L3 larvae and found to produce high protection (69.5%) over the control with multiple types of protective immune responses, thus postulating as a potential vaccine candidate [53]. But our approach is to look at salivary gland protein-derived epitopes as immunogens. The presumption is that antibodies to these proteins would interfere with vector transmission of viruses.

Dimitrov et al. [54] reported a systematic platform for prediction of immunogenicity and MHC class I binding affinity. The platform integrates three quasi-independent modular servers: VaxiJen for immunogenicity and EpiJen to predict peptide binding to MHC class I proteins. We however used MHC prediction first to identify as many epitopes as possible followed by screening and selection of epitopes that survive proteasomal cleavage and TAP efficiency.

The transporter associated with antigen processing (TAP) has an important role in the transportation to the endoplasmic reticulum of the peptide fragments of the proteolysed antigenic or selfaltered proteins. This happens with the association of these peptides with major histocompatibility complex (MHC) class I molecules. The prediction of TAP-binding peptides is highly helpful in identifying the MHC class I-restricted Tcell epitopes and hence in the subunit vaccine designing [55]. In silico prediction comprising of MHC class-I binding, TAP prediction and proteasomal cleavage has been validated previously by in vitro experiments indicating its efficiency and usefulness [56].

Presently, peptide based "designer vaccine" has got substantial attention for its significant role in vaccine design. There are innumerous bioinformatic programs available to design B- and Tcell epitopes and online server programs to model the protein with their own benchmark scores. Therefore, it is important to choose appropriate database that are widely used relevant to the study and more importantly to use more than one tool and compare for the best results.

As the vector control alone is not the most effective measure to prevent the transmission of mosquito-borne diseases, studies on the human antibody response against the mosquito salivary proteins would provide new biomarkers for a direct and accurate evaluation of the human exposure to mosquito bites, at community and individual levels. Salivary biomarkers are now studied for its development and applications to control Aedes- and Anopheles-borne diseases [57].

Web-based immunological bioinformatics resources for T- cell epitope prediction, are reported to be very accurate for most of the identified MHC alleles. To improve these predictions, integrating proteasome cleavage (in conjugation with transporter associated with antigen processing (TAP) binding) prediction and population coverage are suggested. However, MHC class II restricted epitope predictions were shown to display relatively low accuracy compared to MHC class I [58]. In our study, we carried out a comprehensive analysis in search of only T- cell epitopes along with prediction of its ability to survive proteasomal cleavage, TAP efficiency, and immunogenicity along with the population coverage in terms of HLA distribution for the design of novel vaccine candidates. Previously published literature shows the experimental evidence for protective effect of antibodies to certain salivary proteins in susceptible hosts [41]. Also, measurement of such antibodies could indicate extent of mosquito vector activity in the community and form the basis for potential vaccine trial [57]. The findings of this study could be evaluated in appropriate mouse models for flaviviruses and togaviruses infection. Information could also be obtained by screening previously exposed populations with ELISA for antibodies to B-cell epitope from D7L and D7S and gammainterferon assays for response to T-cell epitopes. Measurement of this nature would indicate the activity of the mosquito vectors. This approach will open up need for detailed studies in endemic populations with vector and virus activity.

## Conclusion

Thus we have identified the following two B cell epitopes "LAALHVTAAPLWDAKDPEQF" and "TSEYPDRQNQIEELNKLCKN" from D7L and D7S respectively and also two T cell epitopes "MTSKNELDV" and "YILCKASAF" from D7L and D7S respectively with affinity to the predominant MHC class-I supertypes. We postulate that these epitopes identified could be evaluated towards a potential human vaccine for a plethora of flaviviruses and togaviruses like Dengue, Zika viruses, Chikungunya.

## Figures and Tables

**Table 1:** List of epitopes identified from D7L by both LBtope and ABCpred program

B-cell epitopes	SVM score	Percent Probability of correct prediction	ABCpred score
LAALHVTAAPLWDAKDPEQF	1.068752	85.63	0.76
ECIFKGLRYMTSKNELDVDE	1.065985	85.53	0.73
SVFMHCEALNYPKGSPQRKD	0.859348	78.64	0.84
RAILFGKGESSKKYYQEKGI	0.670824	72.36	0.79
KGSPQRKDLCEIRKYQMGSG	0.653593	71.79	0.76
APLWDAKDPEQFRFITSRCM	0.637916	71.26	0.83
TNDFKEAFDYREVRSKDYFA	0.615075	70.5	0.77
LGWKLEPSDDQATQCYTKCV	0.611205	70.37	0.94
YREVRSKDYFAALTGKLKPY	0.584045	69.47	0.84
CLMNDSKVTNDFKEAFDYRE	0.582745	69.42	0.89
YMTSKNELDVDEIARDFIEV	0.466239	65.54	0.77
ALTGKLKPYSRSDVRKQVDD	0.450293	65.01	0.72
CEIRKYQMGSGIVFGRHMEC	0.374745	62.49	0.82
GSGIVFGRHMECIFKGLRYM	0.347294	61.58	0.73
GIKIKQKGQSVFMHCEALNY	0.335518	61.18	0.83

**Table 2:** List of epitopes identified from D7S by both LBtope and ABCpred program

B-cell epitopes	SVM score	Percent Probability of correct prediction	ABCpred score
TSEYPDRQNQIEELNKLCKN	0.74	74.67	0.69
KNIYDPPRNAMNYLDCIALR	0.51	66.92	0.86
FIRKREPKFFNVFHCRGITL	0.41	63.6	0.9

**Table 3:** T cell epitopes predicted from D7L by BepiPred MHC-I prediction program

T cell epitopes	Specific HLAs	Percentile rank	Start	Length	MAPPP score	TAP score	Immunogenicity score
MFPPRKFLL	HLA-A*23:01	0.6	1	9	1	4.04*	-0.07
	HLA-A*24:02	0.75					
ALHVTAAPLW	HLA-B*58:01	0.7	16	10	1	-	0.15
DAKDPEQFR	HLA-A*33:01	0.3	26	9	0.7351	4.231*	0.006
	HLA-A*68:01	0.8					
ITSRCMEDW	HLA-B*57:01	0.2	36	9	1	7.001#	-0.12
YPKAKNPKAA	HLA-B*08:01	0.9	45	10	0.999	-	-0.42
KAALQNWLGW	HLA-B*57:01, HLA-B*58:01	0.2	52	10	1	-	0.1
AALQNWLGW	HLA-B*57:01, HLA-B*58:01	0.3	53	9	0.99	3.950*	0.09
	HLA-B*44:02	0.85					
KQKGQSVFM	HLA-A*30:01	0.7	174	9	0.5	3.950*	-0.2
	HLA-B*15:01	0.8					
RYMTSKNEL	HLA-A*24:02	0.6	225	9	0.5	3.859*	-0.32
	HLA-A*23:01	0.65					
RYMTSKNELD	HLA-A*24:02	0.65	225	10	0.999	-	-0.32
MTSKNELDV	HLA-A*01:01	1	227	9	0.56	8.221#	-0.18
IEVKKKPDAL	HLA-B*40:01	0.6	243	10	0.79	-	-0.59
VTNDFKEAF	HLA-A*32:01	0.5	285	9	0.5	8.264#	0.03

#Transport associated protein (TAP) binding with high affinity, *Transport associated protein binding with intermediate affinity							

**Table 4:** T cell epitopes predicted from D7S by BepiPred MHC-I prediction program

T cell epitopes	Specific HLAs	Percentile rank	Start	Length	MAPPP score	TAP score	Immunogenicity score
ATILIFSVF	HLA-B*15:01	0.3	4	9	0.53	3.841*	0.16
	HLA-A*26:01	0.4					
	HLA-A*32:01	0.7					
ATILIFSVFV	HLA-A*02:06	0.95	4	10	0.61	-	0.22
ILIFSVFVA	HLA-A*02:01	0.6	6	9	0.99	7.773#	0.16
VASIKTKGIY	HLA-A*30:02	0.3	13	10	0.5	-	-0.19
ASIKTKGIY	HLA-A*30:02	0.2	14	9	0.55	3.842*	-0.23
DYILCKASAF	HLA-A*23:01	0.4	38	10	0.5	-	-0.28
	HLA-A*24:02	0.6					
YILCKASAFL	HLA-B*08:01	0.6	39	10	0.999	-	-0.28
YILCKASAF	HLA-B*08:01	0.6	39	9	0.52	8.176#	-0.35
	HLA-B*15:01	1					
PPRNAMNYL	HLA-B*07:02	0.4	115	9	0.99	8.373#	-0.12
STKEIIPFI	HLA-A*30:01	0.7	132	9	0.51	4.238*	0.34
PFIRKREPKF	HLA-A*23:01	0.65	138	10	0.51	-	-0.1
FIRKREPKF	HLA-B*08:01	0.8	139	9	1	3.887*	-0.19

#Transport associated protein (TAP) binding with high affinity, *Transport associated protein binding with intermediate affinity							

**Table 5:** Evaluation of protein structure models generated for D7L and D7S salivary proteins of Ae. aegypti

Program for protein 3D structure analysis	D7L			D7S		
Template	ERRAT quality factor	Verify 3D	Template	ERRAT quality factor	Verify 3D
I-TASSER	3DX1A	90.741	95.18%	3DYE	93.33	63.92%
Swiss-model	SDYE1A	98.684	91.67%	3DZT	96.72	91.54%
Phyre2	3DZT	84.859	94.92%	3DZT	78.99	53.54%
RaptorX	3DX1A	66.044	88.86%	3DXLA	65.1	53.16%

This analysis of the proteins was carried out by four modeling programs to examine the protein folding which forms the basis for epitope presentation especially to B cells. The Swiss-model compared to other three models (I-TASSER, Phyre2 and Raptor X), had high ERRAT quality factor and passed the Verify_3D score. This validates that the protein-derived epitopes under scrutiny are likely to be potent immunogens.
